# *Toxoplasma gondii* induces MST2 phosphorylation mediating the activation of hippo signaling pathway to promote apoptosis and lung tissue damage

**DOI:** 10.1016/j.isci.2024.111312

**Published:** 2024-11-04

**Authors:** Kangzhi Xu, Shifan Zhu, Fan Xu, Jin Yang, Bin Deng, Dingzeyang Su, Jing Ma, Mingyue Zu, Yifan Lin, Tianxu Pei, Yuyang Zhu, Lele Wang, Dandan Liu, Qiangde Duan, Jinjun Xu, Zhiming Pan, Jianping Tao, Zhaofeng Hou

**Affiliations:** 1 College of Veterinary Medicine, Yangzhou University, Yangzhou 225009, China; 2 Jiangsu Co-innovation Center for Prevention and Control of Important Animal Infectious Diseases and Zoonosis, Yangzhou 225009, China; 3 International Research Laboratory of Prevention and Control of Important Animal Infectious Diseases and Zoonotic Diseases of Jiangsu Higher Education Institutions, Yangzhou University, Yangzhou 225009, China; 4 Jiangsu Key Laboratory of Zoonosis, Yangzhou 225009, China; 5 Northern Jiangsu People's Hospital Affiliated to Yangzhou University, Yangzhou 225000, China; 6 Department of Gastroenterology, Affiliated Hospital of Yangzhou University, Yangzhou 225000, China

**Keywords:** Molecular biology, Microbiology

## Abstract

*Toxoplasma gondii* (*T. gondii*) is an intracellular parasite, and its regulation of host cell apoptosis directly affects its parasitism. Studies link *T. gondii*-induced apoptosis to abnormal expression of mammalian STE20-like protein kinase 2 (MST2), but its precise role remains unclear. In this study, the regulatory roles in apoptosis and pathogenicity of *T. gondii* infection were identified *in vitro* and *in vivo*. Simultaneously, MST2 and Hippo signaling pathway activation induced by *T. gondii* were evaluated. MST2 overexpression and knockout were used to assess its regulatory role in apoptosis and Hippo signaling pathway. Results showed that *T. gondii* induced apoptosis and lung damage, with Hippo signaling pathway activation via MST2 phosphorylation. MST2 was demonstrated to regulate apoptosis and Hippo signaling pathway. Notably, MST2 knockout hindered the *T. gondii*-induced apoptosis and weakened Hippo signaling pathway activation. MST2 is an important target for *T. gondii* to control host cell fate and modulate immune responses.

## Highlights

• *T. gondii* promotes apoptosis and lung damage by inducing MST2 phosphorylation
• MST2 promotes apoptosis and regulates the Hippo signaling pathway
• MST2 knockout inhibits *T. gondii* replication in PK-15 cells

## Graphical abstract

**Figure:**
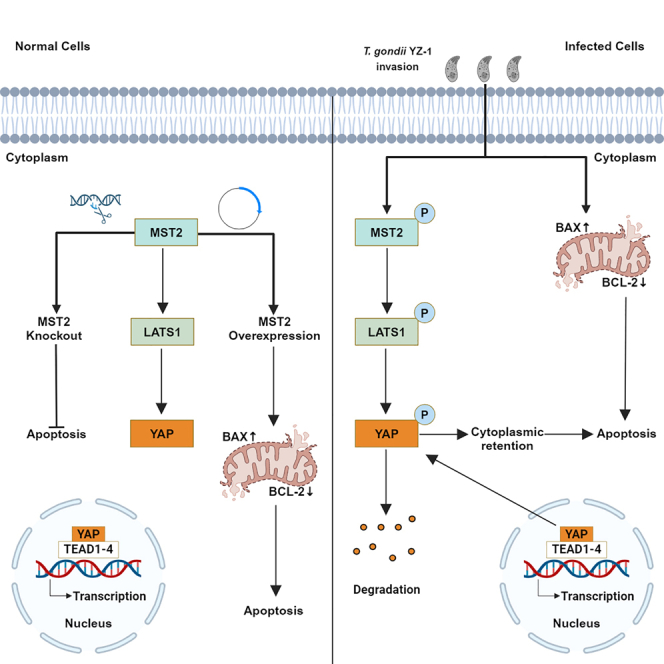

## Introduction

*T. gondii* is an obligate intracellular parasitic protozoan that can cause geographically widespread zoonosis.^1^ Infection in the population is mostly asymptomatic; however, toxoplasmosis is linked to a range of behavioral alterations,^2^ and a poor prognosis usually occurs in newborns and immunosuppressed individuals that are exposed to infection. Human toxoplasmosis outbreaks are correlated with the consumption of raw or undercooked meat, especially from infected pigs or wild boars,^3^^,^^4^ attributing 12% to this disease burden.^5^ Therefore, it is important to control pig toxoplasmosis for maintaining public health, food safety, and sustainable development of breeding industry.

Apoptosis, an important physiological immune reaction that is related to the intracellular survival of pathogens, has been shown to be one of biological processes of the hosts significantly influenced by *T. gondii*.^6^^,^^7^ Prior studies revealed that differentially expressed genes in host cells induced by *T. gondii* invasion were significantly enriched in the Hippo signaling,^8^ which has been considered as a crucial mediator in oxidative stress and apoptosis.^9^ Mammalian STE20-like protein kinase 2 (MST2), a serine/threonine kinase, holds considerable importance within the upstream Hippo signaling pathway due to its participation in cellular oxidative stress, apoptosis, proliferation, and differentiation.^10^^,^^11^

MST2 activation yields notable pro-apoptotic consequences in instances of tissue injury^12^ and oxidative stress,^13^ which is substantiated by its capacity to regulate mitochondrial depolarization^14^^,^^15^ and influence the functions of apoptosis-related proteins through the initiation of a kinase cascade reaction.^16^^,^^17^^,^^18^^,^^19^ Increasing studies have found that mediating Hippo signaling pathway may be a pivotal strategy by which MST2 dominates cell fate. The pathway mainly consists of a core inhibitory kinase cascade that includes components, such as MST1/2, large tumor suppressor homolog 1/2 (LATS1/2) and Yes-associated protein (YAP)/Tafazzin (TAZ).^20^ MST1/2 activation induces phosphorylation of LATS1/2,^21^^,^^22^ followed by phosphorylation and cytoplasmic retention of YAP/TAZ, which suppresses the pro-proliferative and anti-apoptotic activities, thus facilitates apoptosis. In contrast, when upstream signaling is obstructed, the unphosphorylated YAP/TAZ to migrate into nucleus and bind to transcriptional enhanced associate (TEA) domain transcriptional factor 1–4 (TEAD1-4). This interaction stimulates the activities of downstream transcription factors, thereby initiating the pro-proliferative and anti-apoptotic functions of YAP/TAZ.^23^^,^^24^^,^^25^

*T. gondii* often causes systemic infections, with the lungs being one of the most frequently affected organs.^26^ *T. gondii* infection can lead to diffuse pneumonia in immunocompromised human patients, commonly interstitial pneumonia in mice, showing peribronchial and perivascular infiltrate with increased cellularity in the septum, congestion, and atelectasis.^27^ Moreover, unchecked inflammatory cells, excessive release of pro-inflammatory mediators, deposition of fibrin, lung edema formation, and apoptosis are all key factors contributing to acute lung injury.^28^ However, the expression of the Hippo signaling pathway, which is often activated in response to pathological damage in the body, remains unknown in the context of *T. gondii*-induced lung tissue damage.

Recent evidence demonstrated that *T. gondii* activated Hippo signaling by upregulating LATS1 phosphorylation, consequently causing alterations in the transcriptional profile in endothelial cells, which partly explained the molecular interactions at the parasite-endothelial cell interface.^29^ However, the specific roles of Hippo signaling pathway of the hosts following *T. gondii* infection remains unknown. Our previous studies showed that *T. gondii* induced apoptosis,^7^ which was accompanied by abnormal expression of MST2. Nevertheless, whether MST2 and its mediated Hippo signaling pathway participate in apoptosis processes influenced by *T. gondii* remains unclear. Therefore, the current study dissected the potential molecular mechanisms by which *T. gondii* induces apoptosis in host cells via MST2-mediated Hippo signaling pathway. This will expand insights into the regulatory mechanisms of apoptosis in host cells infected with *T. gondii* and further support a considered idea that Hippo signaling may be a desirable target for gene therapy.

## Results

### Sequence analysis of porcine *MST2* gene

**Figure 1:**
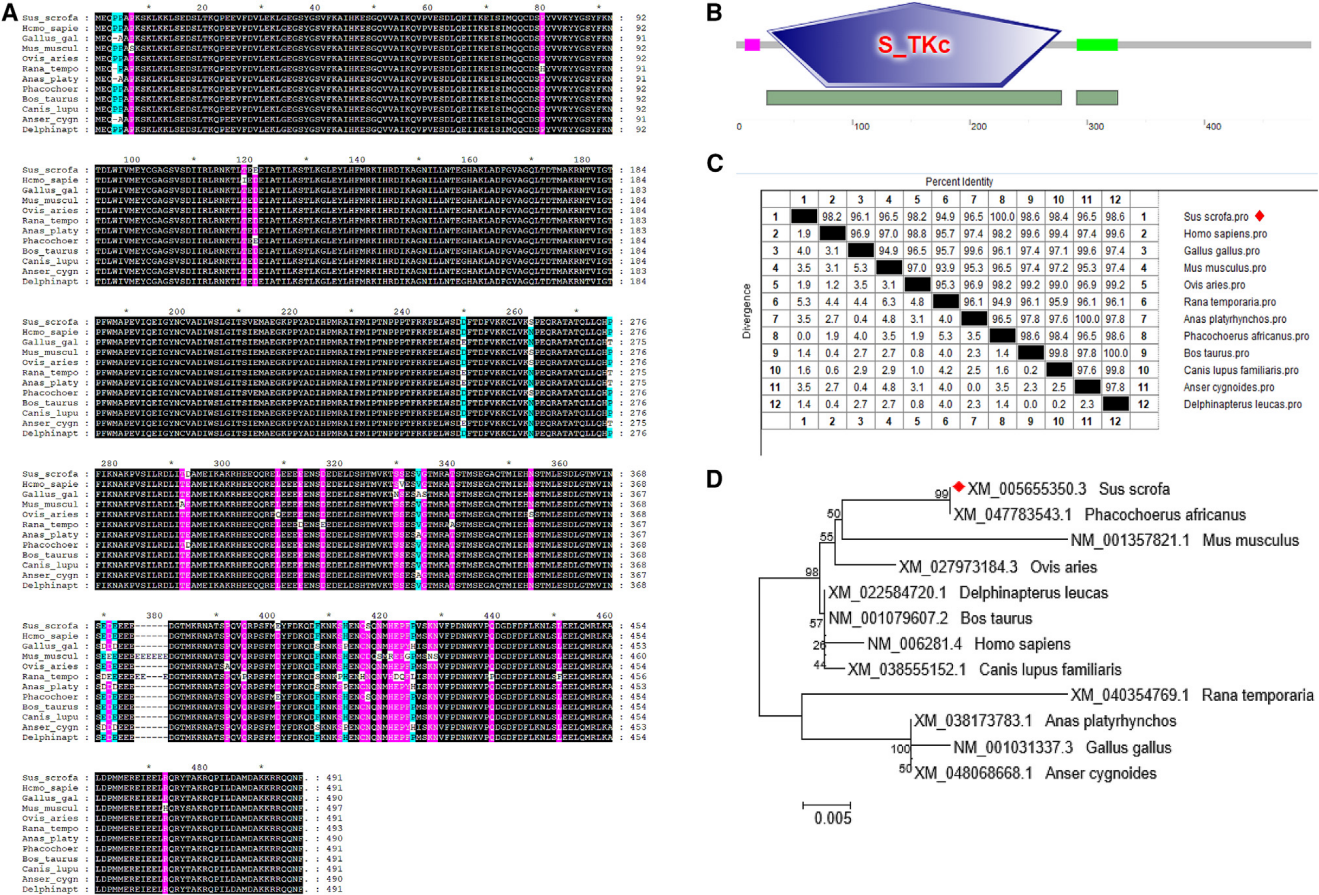
Characteristics of MST2 (A) Use GeneDoc software to compare the MST2 amino acid sequences of pigs, humans, chickens, mice, sheep, and other animals. Black, sequences conserved across the species; purple, protein sequence homology of ≥80%; light blue, protein sequence homology of ≥60%. (B) Prediction of protein structural domains using the SMART program. The pink bars indicate low-complexity domains, located at amino acids 8–21 and the brilliant green bar indicate a coiled coil domains located at amino acids 291–326 of MST2. (C) Sequence distance analysis of MST2 from different species. The analysis was performed with MegAlign software. (D) Phylogenetic tree of the MST2 amino acid sequence and other species sequences. The neighbor joining tree was generated using MEGA-7.

Full-length cDNA of porcine *MST2* is 1,476 bp and encodes 491 amino acids (Figure 1A), in which a serine/threonine protein kinase (S_TKc) structural domain (amino acids 27–278) is contained (Figure 1B**)**. Multiple-sequence alignment reveals that *MST2* is relatively conserved among mammals and birds, and a same phosphorylation site (Thr180) of *MST2* were showed between swine and the other species. The amino acid sequences of *MST2* shares 100%, 98.6%, 98.4%, 98.2%, and 96.1% identities with the counterparts in warthog, cattle, dog, human, and chicken, respectively (Figure 1C**)**. The phylogenetic tree indicates that MST2 branches with pigs (Figure 1D**)**.

### *T. gondii* inducing apoptosis *in vitro*

**Figure 2:**
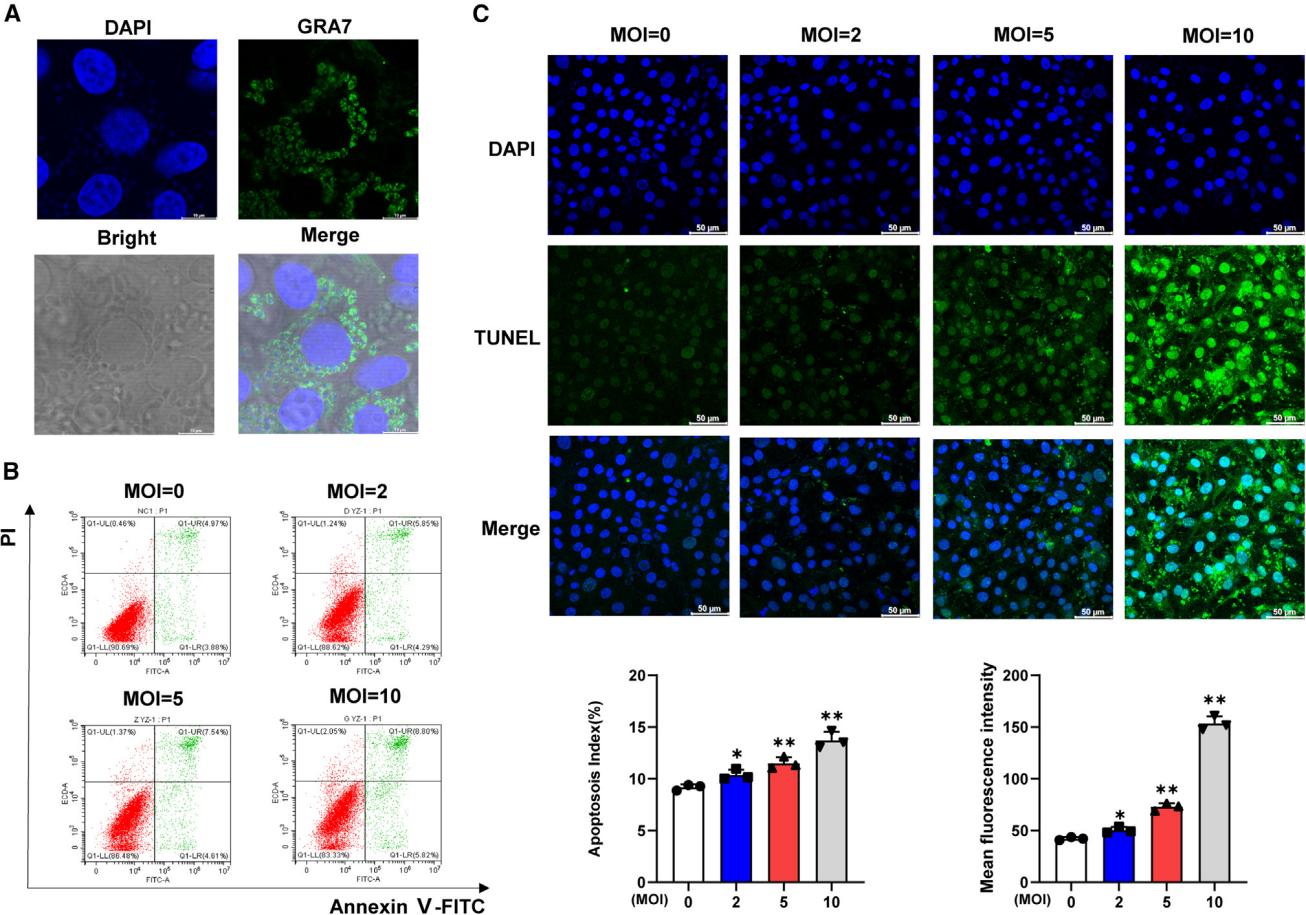
The infection of PK-15 cells with *T. gondii* (MOI = 0, 2, 5, 10) induces apoptosis *in vitro* (A) Detection of YZ-1 tachyzoites infection in PK-15 cells using GRA7 polyclonal antibody, Scale bar, 10 μm. (B) Apoptosis rate of PK-15 cells infected with different doses (MOI = 0, 2, 5, 10) of *T. gondii* for 24 h (*n* = 3). (C) TUNEL staining of PK-15 cells infected with different doses (MOI = 0, 2, 5, 10) of *T. gondii* for 24 h (*n* = 3), scale bar, 50 μm. All graph data are expressed as mean ± SD of at least three biological replicates per group. ∗*p* < 0.05, indicating statistical significance. ∗∗*p* < 0.01, ns, not significant.

A successful model of YZ-1 tachyzoites infection in porcine kidney-15 (PK-15) cells was demonstrated using a mouse-derived dense granule antigen 7 (GRA7) polyclonal antibody by immunofluorescence assay (IFA) (Figure 2A). Flow cytometry (FCM) analysis showed that apoptosis levels of PK-15 cells increased in a tachyzoites dose-dependent manner (Figure 2B). Significant differences were found between the infection and control groups at multiplicity of infection (MOI) = 5:1 or 10:1 (*p* < 0.01). Furthermore, the terminal deoxynucleotidyl transferase dUTP nick end labeling (TUNEL) staining assay provided additional support for this conclusion (Figure 2C).

### *T. gondii* activating the Hippo signaling pathway *in vitro*

**Figure 3:**
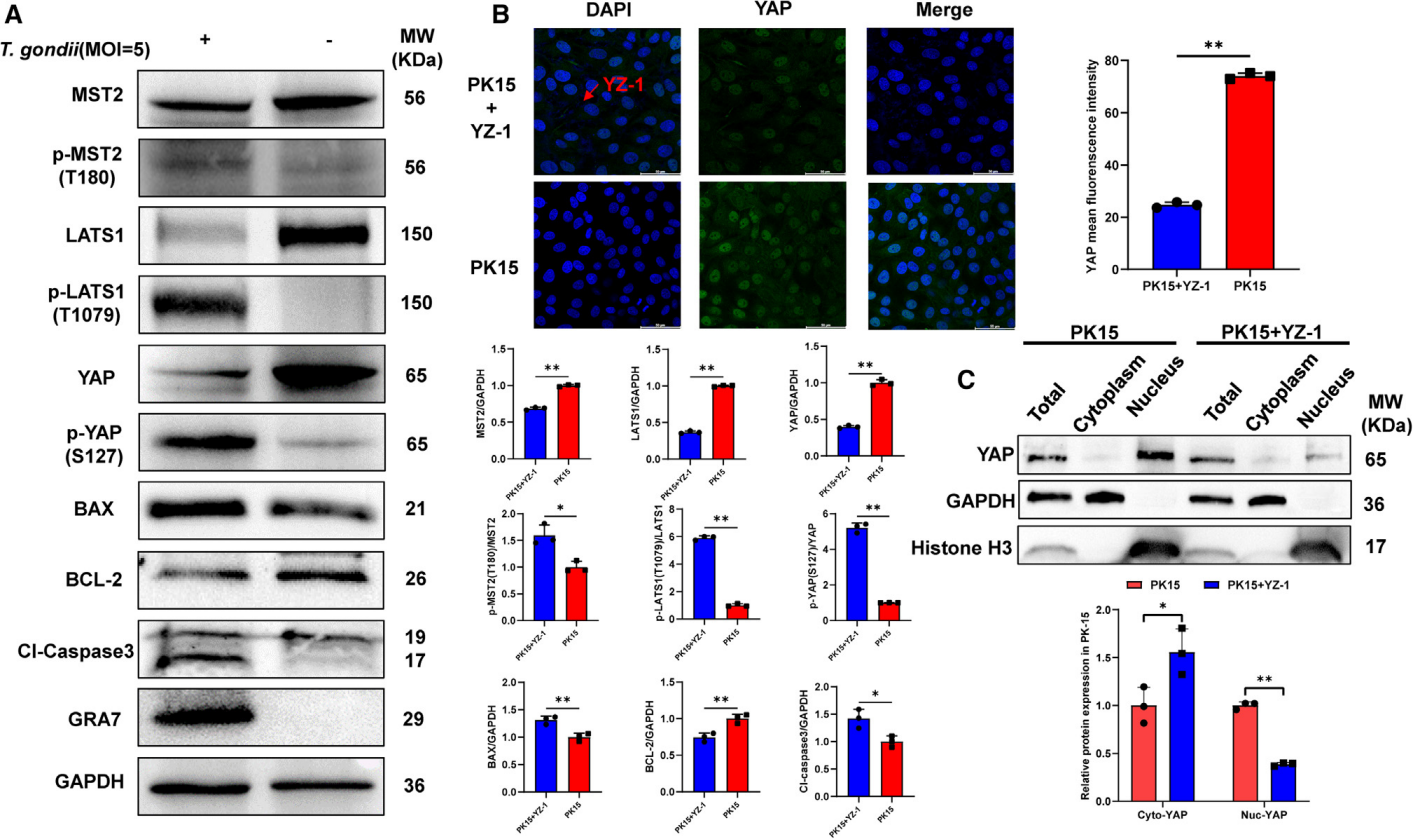
The infection of PK-15 cells with *T. gondii* (MOI = 5) activates the Hippo pathway *in vitro* (A) The differential expression levels of MST2, LATS1, YAP, BAX, BCL-2, and Cl-caspase 3 proteins, as well as their phosphorylated forms p-MST2 (T180), p-LATS1 (T1079), and p-YAP (S127) in PK-15 cells upon infection with *T. gondii* in comparison to uninfected cells. Furthermore, the examination of GRA7 proteins is conducted as potential markers for *T. gondii* infection (*n* = 3). (B) *T. gondii* infection reduces YAP protein expression in the nucleus of PK-15 cells. The red arrow indicates the *T. gondii* of YZ-1 tachyzoites. Scale bar, 50 μm. (C) Cytoplasmic and nuclear fractionation experiments demonstrate that *T. gondii* infection induces the translocation of YAP protein from the nucleus to the cytoplasm. All graph data are expressed as the mean ± SD of at least three biological replicates per group. ∗*p* < 0.05, ∗∗*p* < 0.01, ns, not significant.

To investigate the changes in the expression of the Hippo signaling pathway following *T. gondii* infection, YZ-1 tachyzoites were added to PK-15 cells at an MOI of 5 for 24 h. Western blotting (WB) assay demonstrated that *T. gondii* infection led to a significant reduction in the expression levels of MST2, LATS1, and YAP, while the phosphorylation levels of p-MST2 (T180), p-LATS1 (T1079), and p-YAP (S127) were significantly increased when infected at an MOI of 5:1 for 24 h. The relative expression levels of p-MST2(T180)/MST2, p-LATS1(T1079)/LATS1, p-YAP (S127)/YAP, Bcl-2 Associated X-protein (BAX)/GAPDH, and Cl-caspase3/GAPDH were significantly elevated in *T. gondii*-infected PK-15 cells, while the B-cell lymphoma 2 (BCL-2)/GAPDH was significantly decreased (*p* < 0.05) (Figure 3A). Meanwhile, IFA demonstrated that *T. gondii* infection induced a decreased expression of YAP protein in the nucleus of PK-15 cells (Figure 3B**)**. The results from the nuclear-cytoplasmic fractionation assay further confirmed the translocation of YAP protein from the nucleus to the cytoplasm post-infection (Figure 3C**)**. The aforementioned findings provide the evidences that *T. gondii* infection induces apoptosis and concurrently activates the Hippo signaling pathway in PK-15 cells.

### *T. gondii* causing lung tissue damage and activating Hippo signaling pathway *in vivo*

**Figure 4:**
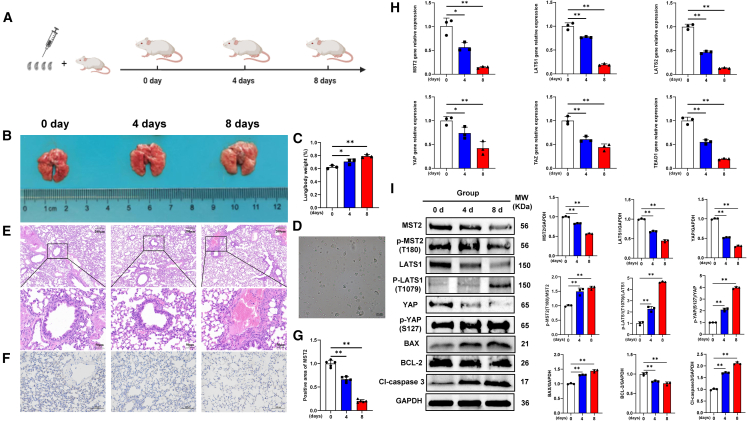
*T. gondii* induces Hippo signaling pathway activation in mouse lung tissue *in vivo* (A) Establishment timeline of *T. gondii*-induced lung injury in a mouse model. (B) Enlargement of lung tissue in mice due to *T. gondii* infection by apparent morphological observation. (C) Lung tissue weight/body weight (*n* = 3). (D)Successful isolation of YZ-1 tachyzoites from the ascites of *T. gondii* infected mice; scale bar, 20 μm. (E) Histopathologic changes in lung tissues of mice at various time points post-infection observed through H&E staining; Scale bar, 200 μm. Insets show a higher magnification of the outlined area. Scale bar, 50 μm. (F) Lung IHC analysis of MST2. Scale bar, 100 μm. (G) positive area of MST2 (*n* = 5). (H) Detection of mRNA expression levels of molecules within the Hippo signaling pathway in the lung (*n* = 3). (I) Detection of the expression levels of Hippo signaling pathway proteins and apoptosis-related proteins in the lung (*n* = 3). All graph data are expressed as the mean ± SD of at least three biological replicates per group. ∗*p* < 0.05, ∗∗*p* < 0.01, ns, not significant.

As illustrated in Figure 4A, *T. gondii* acute infection mouse model was established by us. The mice infected with the *T. gondii* showed a gradual increase in lung tissue size over time (Figure 4B**)** and an increase in lung tissue mass relative to body weight (Figure 4C**)**, and lots of parasites were observed in mouse ascites at 4 and 8 days post-inoculation (DPI) further proved the reliability of the model (Figure 4D). The burden of these parasites caused progressive alterations in the alveolar structure, including widening of alveolar intervals, thinning of alveolar walls, uneven sizes of alveolar cavities, and exacerbation of interstitial congestion. Whereas an absence of discernible damage was found in the structural integrity of the lung tissues in the uninfected mice (Figure 4E). Simultaneously, a decreased expression of MST2 was observed in the infected lung tissues through Immunohistochemistry (IHC) examination (Figures 4F and 4G). Additionally, mRNA expression levels of the key molecules (MST2, LATS1, LATS2, YAP, TAZ, and TEAD1) of Hippo signaling pathway in lung tissues were characterized as significantly decreased (*p* < 0.01) during *T. gondii* infection (Figure 4H), and the protein expression levels of MST2, LATS1, and YAP showed the same results (Figure 4I). However, the significantly increased phosphorylation levels of MST2, LATS1, and YAP in lung tissues were found to be induced by *T. gondii* (Figure 4I). Remarkably, apoptosis may be promoted in the infected lung tissues, as evidenced by the upregulation of BAX, downregulation of BCL-2, and activation of cleaved caspase 3 (Cl-caspase 3) induced by *T. gondii* (Figure 4I). These findings aligned with the outcomes indicating that *T. gondii* infection induced the activation of Hippo signaling pathway *in vivo*.

### MST2 overexpression promoting apoptosis and increasing expressions of downstream effector molecules of Hippo signaling pathway

**Figure 5:**
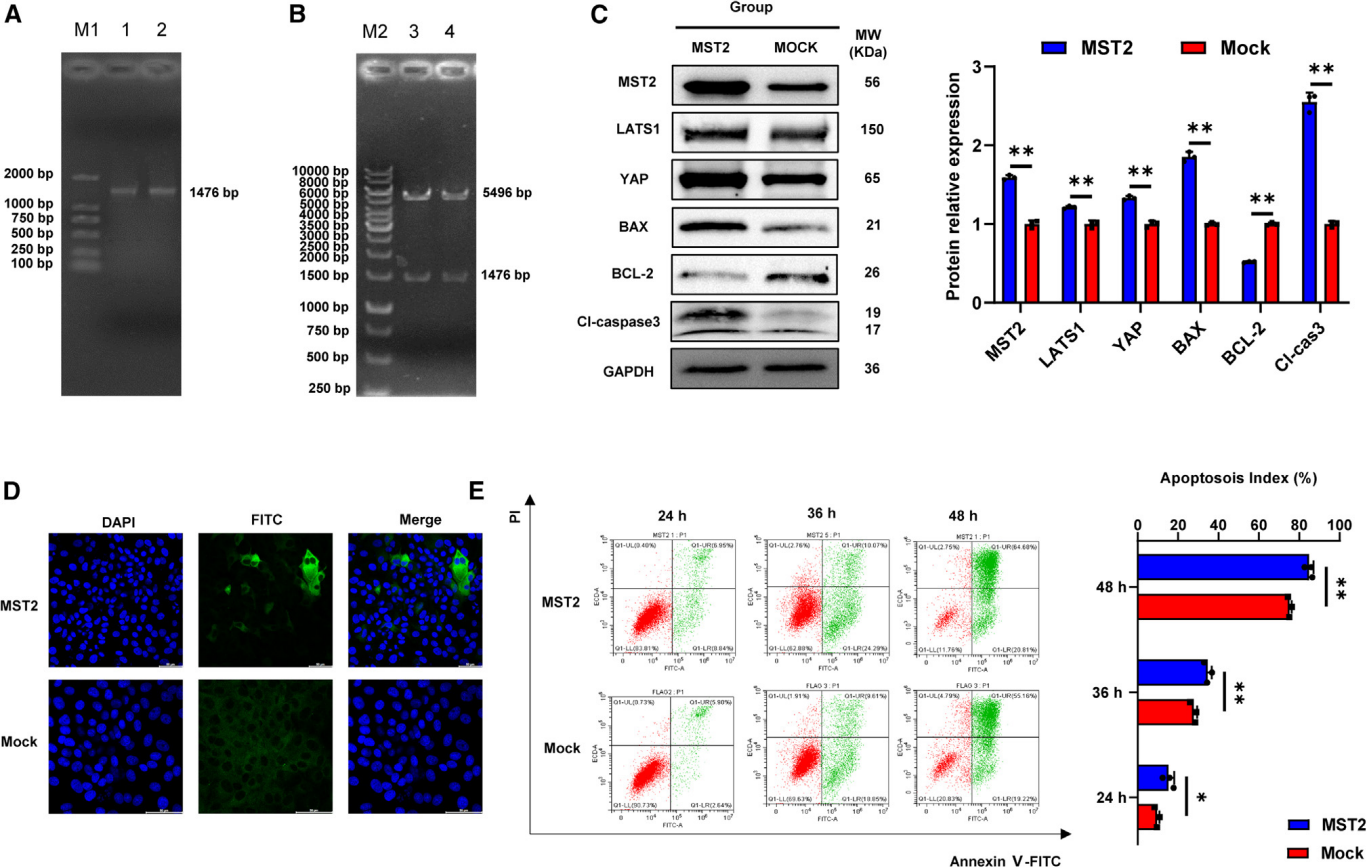
Effects of MST2 overexpression on apoptosis and regulation of the Hippo signaling pathway components LATS1 and YAP (A) Amplification of porcine *MST2* gene. (B) Porcine *MST2* gene overexpression plasmid construction. (C) WB verified successful overexpression of the *MST2* gene and the effect of overexpression of MST2 on LATS1, YAP, BAX, BCL-2, and Cl-caspse 3. (D) IFA experiments showed that MST2 localized in the PK-15 cytoplasm; scale bar, 50 μm. (E) FCM probes the effects of overexpression of MST2 on apoptosis. Abbreviation: MST2: transfection of cells with pcDNA3.1-3FLAG-C-MST2 recombinant plasmid; MOCK: transfection of cells with pcDNA3.1-3FLAG-C plasmid. M1: DL2000 DNA marker; 1,2: results of the MST2 gene amplification. M2: 1kb DNA ladder; 3,4: double digestion was performed on the pcDNA3.1-3FLAG-C-MST2 plasmid. All graph data are expressed as the mean ± SD of at least three biological replicates per group. ∗*p* < 0.05, ∗∗*p* < 0.01, ns, not significant.

Porcine *MST2* gene was effectively amplified with length of 1,476 bp (Figure 5A). And the constructed recombinant overexpression plasmid pcDNA3.1-3FLAG-C-MST2 was confirmed through digestion with *EcoR I* and *Xho I* (Figure 5B). By WB and IFA, MST2 was proved to be successfully overexpressed in PK-15 cells (Figures 5C and 5D), and MST2 overexpression promoted apoptosis by increasing expression of BAX and Cl-caspase 3, while reducing the expression of BCL-2 (Figures 5C and 5E). Furthermore, this study revealed that MST2 can upregulate the expressions of LATS1 and YAP in Hippo signaling pathway (Figure 5C).

### MST2 knockout cell line construction

Three pairs of single guide RNAs (sgRNA79, sgRNA15, and sgRNA8) were specifically designed to target exons 3, 4, and 7 of the *MST2* gene (Figure S1A) and then ligated with the linearized lenti-CRISPRV2 vectors (Figure S1B), respectively. DNA sequencing demonstrated that the recombinant knockout plasmids were successfully constructed (Figure S1C). After drug screening and single-cell expansion, MST2 knockout cells were obtained. DNA sequencing revealed mutations in sgRNA8 (A-base insertion), sgRNA15 (C-base deletion), and sgRNA79 (consecutive deletion of GTTGTGGCAA base) compared to normal PK-15 cells (Figure S1D). All three groups of sgRNAs showed high knockout efficiency (Figures S1E and S1F), among which sgRNA8 manifested the highest knockout efficiency, and was designated as MST2-KO.

### MST2-mediated Hippo signaling pathway involved in *T. gondii* induced apoptosis

**Figure 6:**
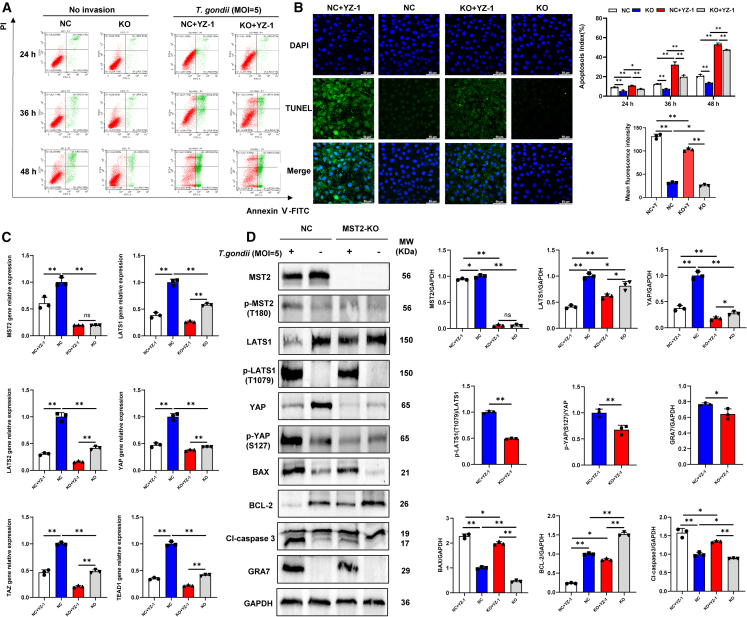
Regulation of apoptosis, Hippo signaling pathway by MST2 knockout under *T. gondii* (MOI = 5) infected and uninfected conditions (A) Apoptosis rate of NC + YZ-1, NC, KO + YZ-1, KO four groups at different time periods (*n* = 3). (B) TUNEL staining of NC + YZ-1, NC, KO + YZ-1, KO four groups at MOI = 5 of *T. gondii* for 24 h (*n* = 3); scale bar, 50 μm. (C) The mRNA expression levels of Hippo signaling pathway-related molecules in the four groups of NC + YZ-1, NC, KO + YZ-1, KO (*n* = 3). (D) The expression levels of Hippo signaling pathway-related proteins and apoptosis-related proteins were measured in the four groups of NC + YZ-1, NC, KO + YZ-1, KO (*n* = 3). Abbreviation: NC: normal PK-15 cells; KO: MST2 knockout PK-15 cells of the sgRNA8 group. All graph data are expressed as the mean ± SD of at least three biological replicates per group. ∗*p* < 0.05, ∗∗*p* < 0.01, ns, not significant.

After administering equivalent doses of *T. gondii* (MOI = 5) to both normal and MST2-KO cell groups, FCM results indicated a significant inhibition of apoptosis in PK-15 cells following MST2 knockout, as well as the suppression of apoptosis induced by *T. gondii* infection (Figure 6A). Additionally, the TUNEL staining assay further confirmed these findings (Figure 6B). Real-time quantitative PCR (real-time qPCR) and WB analysis demonstrated that MST2 knockout not only led to a significant reduction in the mRNA expression levels of LATS1/2, YAP/TAZ, and TEAD1, and the protein expression levels of LATS1 and YAP in normal cells, but a significant reduction in the activation of Hippo signaling pathway was also observed in the MST2-KO cells following *T. gondii* infection at 24 h. This was further supported by the lower expression levels of p-LATS1 (T1079)/LATS1, p-YAP (S127)/YAP in the *T. gondii*-infected MST2-KO cells compared to the normal cells. At the same time, under both *T. gondii*-infected and uninfected conditions, MST2 knockout leads to a decrease in the expression levels of BAX and Cl-caspase 3 proteins and an increase in the expression of BCL-2 protein compared to normal cells (Figures 6C and 6D). These results indicate that MST2-mediated Hippo signaling pathway was involved in *T. gondii* induced apoptosis in PK-15 cells.

### MST2 knockout inhibits *T. gondii* replication in PK-15 cells

**Figure 7:**
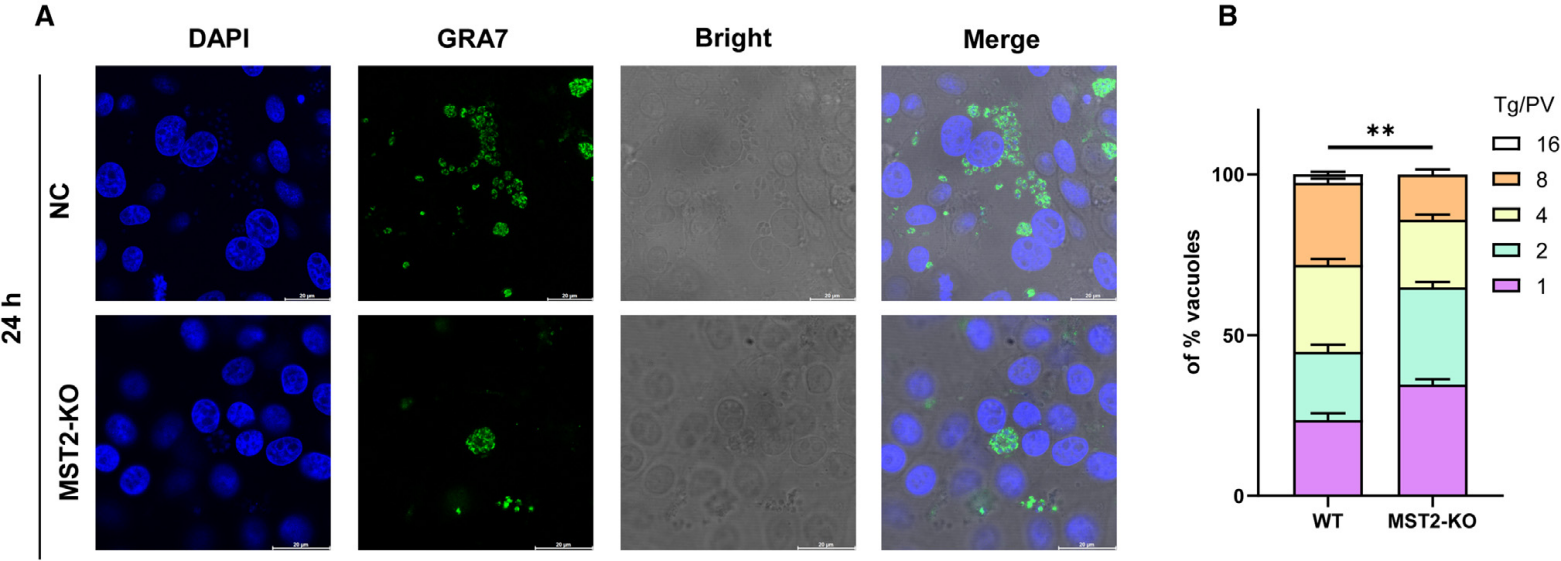
Effect of MST2 gene knockout of PK-15 cells on the *T. gondii* intracellular proliferation (A) IFA assay investigating the impact of MST2 knockout on tachyzoite replication; scale bar, 50 μm. (B) Intracellular proliferation assay exploring the effect of MST2 knockout on tachyzoite replication. Abbreviation: Tg: *T. gondii* YZ-1 tachyzoites; PV: parasitophorous vacuole; 1, 2, 4, 8, and 16 represent the number of tachyzoites in each PV. All graph data are expressed as mean ± SD of at least three biological replicates per group. ∗*p* < 0.05, ∗∗*p* < 0.01, ns, not significant.

After 24 h of infection with *T. gondii* (MOI = 5), the WB results indicated that the expression level of GRA7 protein in MST2-KO cells was lower than that in wild-type PK-15 cells (Figure 6D). Furthermore, the intracellular proliferation assay showed that after infecting PK-15 and MST2-KO cells with 1,000 YZ-1 tachyzoites for 24 h, the maximum number of tachyzoites in the parasitophorous vacuole (PV) of MST2-KO cells was 8, while in wild-type PK-15 cells, the number reached up to 16. Additionally, the number of PV containing one or two tachyzoites was predominantly observed in MST2-KO cells, while vacuoles with four or eight tachyzoites were more commonly seen in wild-type PK-15 cells (Figures 7A and 7B). Taken together, the data imply that MST2 is necessary for the replication of *T. gondii* in PK-15 cells.

## Discussion

Apoptosis has been shown to be one of the biological processes significantly influenced by *T. gondii*, whose capacity and outcome to modulate the cellular apoptotic machinery were affected by virulence and loads, as well as the host cell types.^30^^,^^31^^,^^32^^,^^33^ BAX and BCL-2 are key factors in apoptosis. Upregulation of BAX and downregulation of BCL-2 can lead to mitochondria-dependent caspase activation, thereby inducing apoptosis. Caspase acts as an executor of apoptosis, and once activated, it produces cleaved forms, leading to cell death.^34^ *Caspase-3* is a hub gene in the apoptosis pathway and plays a pivotal role in apoptotic execution.^35^ Prior researches demonstrated that *T. gondii* triggered caspase-3 activation in mouse neural stem cells and human umbilical cord mesenchymal stem cells (hUC-MSCs),^36^^,^^37^ which is primarily characterized by an elevation in its cleaved form (Cl-caspase 3) expression. This study showed that *T. gondii* triggers apoptosis in PK-15 cells, with the rate of apoptosis positively correlated with infection dose, suggesting that rapid proliferation of tachyzoites manifested a significant pathogenic effect on PK-15 cells. Meanwhile, infection *in vivo* further confirmed the strong pathogenic effect of *T. gondii* by inducing apoptosis that Cl-caspase 3 was synchronously activated by *T. gondii* in mouse lung tissues and PK-15 cells.

*T. gondii*-induced pro-apoptotic effects were involved in a variety of cell types, including hUC-MSCs, Vero, MLTC-1, human leukemia T cells, and mouse spleen tissues as previous studies.^8^^,^^37^^,^^38^^,^^39^^,^^40^ While more studies have found that *T. gondii* maintains its intracellular parasitism and modifies host immune responses through inhibiting apoptosis, which is particularly significant during the introduction of apoptosis inducers, including Fas-induced cytotoxicity, IL-2 deprivation, irradiation, UV, calcium ionophores, and beauvericin.^41^ These regulation strategies by *T. gondii* may depend on infection backgrounds and cell microenvironments. However, often of interest is whether such induced alterations in cell fate are dominated by the host cells. Factually, autonomously initiating apoptosis is a key immune defense mechanism for the hosts to control the intracellular pathogens.^42^^,^^43^ The alteration of apoptosis programs is an important competitive mechanism between *T. gondii* and the host. Investigation across the board seemed to show that it benefits only one side under a given context of infection. Even so, understanding the regulatory mechanism of apoptosis is still crucial for the development of the host against *T. gondii* infection. But, an important unanswered question is how the parasite regulates apoptosis processes.

MST2 is a crucial constituent of the evolutionarily conserved Hippo signaling pathway and holds a prominent position in the regulation of cellular development, proliferation, and apoptosis.^44^^,^^45^^,^^46^^,^^47^ It had been demonstrated that MST2 upregulation facilitated apoptosis in pancreatic cancer cells by activating PI3K/AKT/mTOR pathway. Conversely, MST2 downregulation impeded apoptosis.^48^ Consistent with previous studies, our study revealed the pro-apoptotic effect of porcine MST2, indicating that its abnormal expression is a pivotal molecular event for regulating apoptosis processes of PK-15 cells. Moreover, *T. gondii* infection induced phosphorylation of MST2, and during infection, MST2 knockout suppressed *T. gondii*-induced apoptosis. Therefore, MST2 phosphorylation is thought to play a key role in the *T. gondii*-induced apoptosis. Notably, this study provides direct evidence that MST2 possesses the ability to efficiently regulate the expressions of downstream molecules of Hippo pathway, because the synchronized changes were observed in expressions of LATS1 and YAP upon overexpression and knockout of MST2 in PK-15 cells. Additionally, *T. gondii* induced MST2 phosphorylation, thus activated LATS1 and YAP, but MST2 knockout caused significant reduction in the activation of Hippo signaling pathway and Cl-caspase 3 expression during *T. gondii* infection, demonstrating that *T. gondii* activated Hippo signaling pathway by inducing MST2 phosphorylation, which directly involved in the parasite-induced apoptosis.

**Figure 8:**
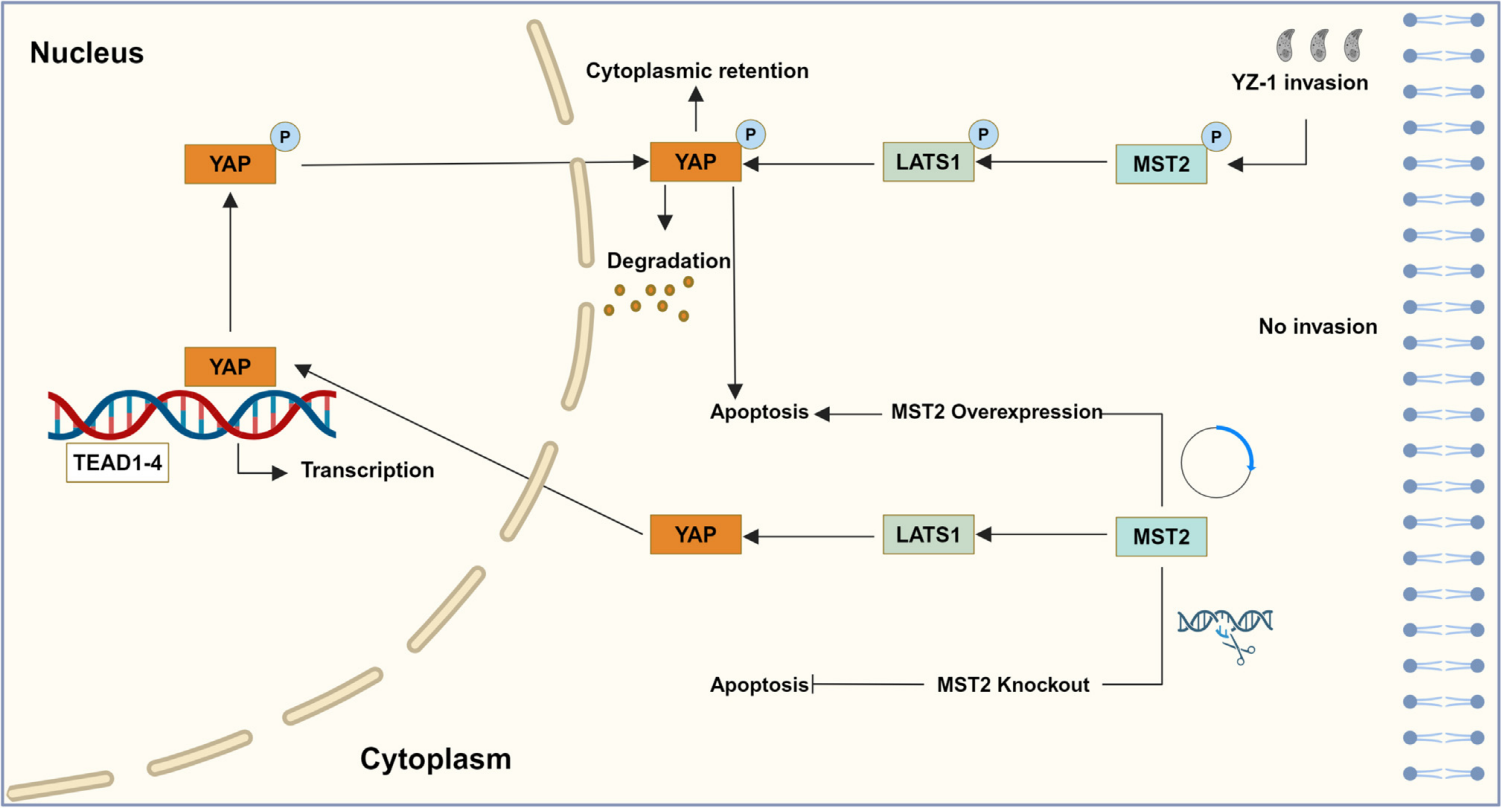
Schematic representation of MST2-mediated Hippo signaling pathway involved in *T. gondii* induced apoptosis in PK-15 cells

Hippo signaling pathway modulates cell proliferation, differentiation, and survival and dysregulation of the pathway is often suggestive of diseases development.^49^^,^^50^ Hippo pathway is activated by sodium arsenite in PC12 cells, along with an increase in apoptosis, while blocking activation reduces apoptosis.^51^^,^^52^ Mice lacking Yap/Taz in alveolar epithelial type II cells exhibited prolonged inflammatory responses and delayed in alveolar epithelial regeneration during bacterial pneumonia.^53^ Thus far, only limited studies have been conducted on the interactions between *T. gondii* and Hippo signaling pathway. *T. gondii* infection (MOI = 2) has been found to induce modifications in the expressions of Hippo pathway-associated genes in endothelial cells. Specifically, there is an increase in LATS1 phosphorylation, and a facilitation of the translocation of YAP from nucleus to cytoplasm, but the overall levels of LATS1 and YAP remain unaffected.^29^ In this study, *T. gondii* triggered the activation of Hippo signaling pathway *in vivo* and *in vitro*, as evidenced by decreased expressions in overall levels of MST2, LATS1, and YAP, accompanied by increased their phosphorylation levels. This phenomenon bears resemblance to the role of chaetocin as a stimulator of Hippo signaling pathway and inducer of apoptosis in esophageal squamous carcinoma cells.^54^ MST2-mediated Hippo signaling pathway contributing to *T. gondii*-induced apoptosis and pathological damages in lung tissues of the hosts has been rationally and favorably supported by this study. Additionally, further investigation revealed that compared to the intracellular replication speed of YZ-1 tachyzoites in PK-15 wild-type cells, the intracellular replication speed of YZ-1 tachyzoites was significantly reduced in MST2-KO cells. This may reveal a mechanism by which *T. gondii* causes pathology and regulates apoptosis (Figure 8). Together with the previous study, the current findings indicate that the activation of Hippo signaling pathway induced by *T. gondii* may be relatively conserved across species and cell types, suggesting that this pathway has excellent potential for the development of drug targets for toxoplasmosis.

### Conclusion

The current results provide an important and infrequent evidence that *T. gondii* has a significant pathogenicity on swine-derived cells through promoting apoptosis. *T. gondii* significantly enhanced the phosphorylation level of MST2 and thus activated Hippo signaling pathway. MST2 knockout not only significantly hindered *T. gondii*-induced apoptosis of host cells but also led to weakened activation of Hippo signaling pathway. It was further discovered that MST2-KO inhibited the intracellular proliferation of *T. gondii*. Therefore, MST2-mediated Hippo signaling pathway contributes to the regulation of apoptosis processes induced by *T. gondii*. MST2 may be an important target for *T. gondii* to control cell fate and modify immune responses of the hosts.

### Limitations of the study

The present study demonstrates that *T. gondii* YZ-1 of porcine origin can lead to PK-15 apoptosis and lung tissue damage in mice by inducing host MST2 phosphorylation and thereby activating the host Hippo signaling pathway. A limitation of this study is that it was not investigated what molecules specifically in *T. gondii* regulate this process of host MST2 phosphorylation. We have demonstrated that PK-15 cells knocked out for MST2 were attenuated to *T. gondii* induced host MST2 protein phosphorylation, while the protective effect of inhibiting MST2 protein expression *in vitro* on the mouse organism was not explored. Therefore, further studies are needed to investigate the molecular mechanism of *T. gondii* YZ-1 interaction with host MST2 protein.

## Resource availability

### Lead contact

Further information and requests for resources and reagents should be directed to and will be fulfilled by the lead contact, Zhaofeng Hou (zfhou@yzu.edu.cn).

### Materials availability

This study did not generate new unique reagents.

### Data and code availability

• The datasets generated and analyzed related to this paper are available from the lead contact on reasonable request.
• This paper does not report original code.
• Any additional information required to reanalyze the data reported in this paper is available from the lead contact upon request.

## Acknowledgments

The authors are grateful to the participants in this study and the anonymous reviewers and editors for their comments and valuable inputs. Additionally, we especially thank the Testing Center of Yangzhou University for providing technical support. This study was funded by the 10.13039/501100001809National Natural Science Foundation of China (no. 32373041 and 32002303), the 10.13039/501100002858China Postdoctoral Science Foundation (no. 2020M671615), the International Research Laboratory of Prevention and Control of Important Animal Infectious Diseases and Zoonotic Diseases of Jiangsu Higher Education Institutions (no. 6), the 10.13039/501100005145Basic Research Program of Jiangsu Province (no. BK20190885) to Z.F.H., the Science and Technology Projects of Yangzhou City (no. YZ2022092) to J.Y., the Postgraduate Research & Practice Innovation Program of Jiangsu Province (no. SJCX23_2009) to K.Z.X., the Undergraduate Innovation and Entrepreneurship Training Program of Yangzhou University (no. X20220678to Y.Y.Z. and C202311117008Y to T.X.P.), and a project funded by the 10.13039/501100012246Priority Academic Program Development of Jiangsu Higher Education Institutions (PAPD). The funders had no role in study design, data collection and analysis, decision to publish, or preparation of the manuscript.

## Author contributions

Conceptualization, J.T. and Z.H.; Data curation, K.X., S.Z. F.X., J.Y., B.D., and D.S.; funding acquisition, Z.H., J.Y., K.X., T.P., and Y.Z.; methodology, J.M., M.Z., Y.L., T.P., Y.Z., and L.W.; resources, D.L., Q.D., J.X., and Z.P.; writing—original draft, K.X.; writing—review and editing, J.T. and Z.H. All authors have read and agreed to the published version of the manuscript.

## Declaration of interests

The authors declare that they have no competing interests.

## STAR★Methods

### Key resources table

**Table:** 

REAGENT or RESOURCE	SOURCE	IDENTIFIER
**Antibodies**		

Anti-MST2	ABclonal	Cat# A9036 RRID:AB_2863645
Anti-phospho-MST2-T180	ABclonal	Cat# AP1094 RRID:AB_2863963
Anti-GAPDH	ABclonal	Cat# AC002 RRID:AB_2736879
Anti-Histon H3	ABclonal	Cat# A17562 RRID:AB_2770395
Anti-BAX	ABclonal	Cat# A19684 RRID:AB_2862733
Anti-BCL-2	ABclonal	Cat# A19693 RRID:AB_2862738
HRP-conjugated Goat anti-Rabbit IgG (H + L)	ABclonal	Cat# AS014 RRID:AB_2769854
HRP-conjugated Goat anti-Mouse IgG (H + L)	ABclonal	Cat# AS003 RRID:AB_2769851
Anti-LATS1	Proteintech	Cat# 17049-1-AP RRID:AB_2281011
Anti-phospho-LATS1-T1079	ABclonal	Cat# AP1517 RRID:AB_3662865
Anti-YAP	ABclonal	Cat# A19134 RRID:AB_2862627
Anti-phospho-YAP-S127	Abmart	Cat# T55743 RRID:AB_2927792
Anti-Cleaved-Caspase 3	Cell Signaling Technology	Cat# 9661 RRID:AB_2341188
Anti-GRA7	Zhu et al.^55^	https://link.cnki.net/doi/10.16872/j.cnki.1671-4652.2021.06.010; RRID: AB_3665442
FITC-labeled Goat Anti-Rabbit IgG (H + L)	Beyotime	Cat# A0562 RRID:AB_2923335
FITC-labeled Goat Anti-Mouse IgG (H + L	Beyotime	Cat# A0568 RRID:AB_2893016

**Bacterial and virus strains**		

*E. coli* DH5α	TransGen	CD201
*E. coli* STBL3	TransGen	CD521

**Chemicals, peptides, and recombinant proteins**		

Puromycin Dihydrochloride	Beyotime	ST551
Antifade Mounting Medium with DAPI	Beyotime	P0131
Lipofectamine™ 3000 Transfection Reagent	Thermo Fisher Scientific	L3000015
RIPA Lysis Buffer	New Cell & Molecular	WB3100
Protease and Phosphatase Inhibitor Cocktail	New Cell & Molecular	P002

**Critical commercial assays**		

Annexin V-FITC/PI Apoptosis Detection Kit	Beyotime	C1062
One Step TUNEL Apoptosis Assay Kit	Beyotime	C1088
Nuclear and Cytoplasmic Protein Extraction Kit	Beyotime	P0028
DAB Horseradish Peroxidase Color Development Kit	Beyotime	P0203
FastPure Cell/Tissue Total RNA Isolation Kit	Vazyme	RC112
HiScript III All-in-one RT SuperMix Perfect for qPCR	Vazyme	R333
AceQ Universal SYBR qRT-PCR Master Mix Kits	Vazyme	Q511
TIANamp Genomic DNA Kit	TIANGEN	DP304

**Experimental models: Cell lines**		

PK-15	Shanghai Zhong Qiao Xin Zhou Biotechnology Co., Ltd	ZQ0516

**Oligonucleotides**		

Primers for PCR, qPCR, and sgRNA are shown in Table S1.	This paper	N/A

**Recombinant DNA**		

pcDNA3.1-3Flag-C-MST2	This paper	N/A
lenti-CRISPRV2-sgRNA79,15,8	This paper	N/A

**Software and algorithms**		

GeneDoc	Open source	http://iubioarchive.bio.net/soft/molbio/ibmpc/genedoc-readme.html; RRID:SCR_025963
SMART	Open source	http://smart.embl.de/; RRID:SCR_005026
MegAlign	DNASTAR	https://www.dnastar.com/workflows/multiple-sequence-alignment/; RRID:SCR_011854
MEGA7	Kumar, S. et al.^56^	http://megasoftware.net/; RRID:SCR_000667
CytExpert	Open source	https://www.beckman.fr/flow-cytometry/instruments/cytoflex/software; RRID:SCR_017217
Graph Pad Prism 8.0.2	Open source	http://www.graphpad.com/; RRID:SCR_002798
ImageJ	Open source	https://imagej.net/; RRID:SCR_003070

### Experimental model and subject details

*T. gondii* YZ-1 strain, isolated from a home-bred wild boar in Jiangsu Province of China, is virulent in mice and belongs to ToxoDB #9 (Chinese I genotype),^57^ which is the most prevalent genotype in human and animals of China. Tachyzoites were harvested by collecting ascitic fluid from mice with severe *T. gondii* infections and filtering it through a 3 μm membrane filter (Whatman, Maidstone, UK) to remove other cells from the peritoneal cavity. The tachyzoites were then counted using a hemocytometer for further experiments.

The Porcine kidney-15 (PK-15) cell line, originally sourced from ATCC, was obtained from Shanghai Zhong Qiao Xin Zhou Biotechnology Co., Ltd (Shanghai, China) and cultured in Nunc Easyflasks (Thermo Scientific, Waltham, MA, USA). The cells were maintained in Dulbecco’s Modified Eagle Medium (DMEM, Thermo Scientific, Waltham, MA, USA) supplemented with 10% fetal bovine serum (FBS), 100 U/mL penicillin, and 10 mg/mL streptomycin at 37°C in a 5% CO₂ atmosphere. Additionally, the PK-15 cell line tested negative for mycoplasma contamination.

Fifteen specific-pathogen-free 6-week-old female BALB/c mice were purchased from the Comparative Medicine Center of Yangzhou University (Yangzhou, China). The mice were domesticated in a controlled temperature (25 ± 1°C) and had free access to food and water. Mice were randomly divided into three groups for peritoneal inoculation with 2000 tachyzoites and euthanized at 0, 4, and 8 days post-inoculation (DPI) in order to obtain lung tissues. This experiment was repeated three times. All animals were handled in strict accordance with good animal practice as defined by the Animal Ethics Procedures and Guidelines of the People’s Republic of China. The study protocol was approved by the Animal Care and Use Committee of the College of Veterinary Medicine, Yangzhou University (Approval ID: SCXK [Su] 2021-0013).

### Method details

#### Flow cytometry (FCM)

The apoptotic levels of PK-15 cells were determined following the instruction of Annexin V-FITC/PI Apoptosis Detection Kit. Briefly, PK-15 cells were seeded at a density of 3 × 10⁵ cells per well in a six-well plate. After 12 h, when the cell density reached 60–80%, one well of cells was collected and counted. Fresh *T. gondii* YZ-1 strain tachyzoites were purified from the ascitic fluid of mice and counted. The required number of tachyzoites was then added to the six-well plate for co-culture according to the needs of each experiment. After the desired infection time, cells were harvested and resuspended in 200 μL binding buffer, and were incubated with FITC-conjugated annexin V and propidium iodide at 4°C for 15 min in the dark. The apoptosis rates were analyzed using a CytoFLEX Flow Cytometer (Beckman, Shanghai, China).

#### TUNEL staining

PK-15 cells were seeded at 5×10^4^ cells/well in a 24-well cell culture plate containing coverslips. After 12 h, when the cells density has reached 60–80%, one well of cells was collected and counted. Meanwhile, freshly purified YZ-1 tachyzoites were counted and used to infect the cells. After 24 h of infection, the cells were washed once with phosphate buffered saline (PBS), followed by the addition of 4% paraformaldehyde to fix the cells at room temperature for 30 min. The cells were washed again with PBS, incubated with 0.3% Triton X-100 in PBS for 5 min at room temperature, and washed twice with PBS. Then, 50 μL of TUNEL detection solution was added to the coverslips and incubated at 37°C in the dark for 60 min. After washing three times with PBS, the coverslips were inverted onto slides containing Antifade Mounting Medium with DAPI and fixed. Confocal fluorescence microscopy was performed on a Leica SP8 FALCON microscope (Leica Microsystems, Wetzlar, Germany) equipped with a Leica TCS SP8 X scanner.

#### Real-time quantitative PCR (real-time qPCR)

Total RNA samples were isolated from cells and tissues using FastPure Cell/Tissue Total RNA Isolation Kit, and 1 μg of total RNA was reverse transcribed into cDNAs using HiScript III All-in-one RT SuperMix Perfect for qPCR. The real-time qPCR reactions were performed using the AceQ Universal SYBR qPCR Master Mix with triplicate of each. The relative expression levels of *MST2*, *LATS1*, *LATS2*, *YAP*, *TAZ* and *TEAD1* genes (primer sequences in Table S1) were quantified and normalized to glyceraldehyde-3-phosphate dehydrogenase (GAPDH) gene using the 2^−ΔΔCt^ method.^58^

#### Western blotting (WB)

Total proteins were extracted from cells and tissues using RIPA Lysis Buffer supplemented with protease and phosphatase inhibitor cocktail. Equal amounts 50 μg of proteins were separated on sodium dodecyl sulfate–polyacrylamide gel electrophoresis (SDS-PAGE), and then transferred to polyvinylidene fluoride (PVDF) membranes. The membranes were incubated with primary antibodies overnight at 4°C, washed five times with TBST, following by incubated with HRP-conjugated secondary antibodies for 1 h at room temperature, washed five times with TBST. Protein bands were visualized using an enhanced chemiluminescence reaction buffer and imaged on a Tanon automatic chemiluminescence image analysis system (Tanon, Shanghai, China). Rabbit antibodies including anti-MST2, anti-phospho-MST2-T180, anti-LATS1, anti-phospho-LATS1-T1079, anti-YAP, anti-phospho-YAP S127, anti-BAX, anti-BCL-2 and anti-Cl-caspase 3 at a 1:1 000 dilution; Mouse antibodies including anti-GRA7 and anti-GAPDH at a 1:5000 dilution were used as primary antibodies to detect the corresponding proteins, respectively.

#### Immunofluorescence assay (IFA)

Cells were cultured on cover slips, then fixed with 4% paraformaldehyde, permeabilized with 0.3% Triton X-100, and blocked with 1% bovine serum albumin (BSA). After incubated with mouse anti-GRA7 polyclonal antibody, rabbit anti-MST2 and YAP monoclonal antibodies at a 1:100 dilution overnight at 4°C, the cover slips were incubated with FITC-conjugated goat anti-rabbit IgG at a 1:200 dilution.

#### Hematoxylin and eosin (H&E) staining and immunohistochemistry (IHC)

Lung tissue samples were fixed in 4% paraformaldehyde, dehydrated with gradient xylene and paraffin, and then embedded in paraffin for sectioning. After stained with H&E, slides were scanned and digitized with a Panoramic SCAN, and histopathological lesions were evaluated with CaseViewer 2.3 (3DHISTECH Ltd., Hungary). Meanwhile, tissues were deparaffinized, hydrated in graded ethanol concentration, and were then activated epitopes in a citrate-buffered solution. Endogenous peroxidase activity was quenched with 3% H_2_O_2_ and incubated with BSA to minimize nonspecific binding. Subsequently, the sections were incubated with rabbit anti-MST2 monoclonal antibody and secondary HRP-Goat Anti-Rabbit IgG antibody at 1:100, respectively. Protein immunoreactivity was visualized with the substrate chromogen 3,3N-Diaminobenzidine. After counterstained in hematoxylin, stained sections were imaged under a Nikon Eclipse microscope (Nikon DS-U3, Tokyo, Japan).

#### *MST2* gene cloning and overexpression

Cleavage sites and protective bases were incorporated to design primers (Table S1) for amplifying the coding sequence (CDS) region of *MST2* gene (Genebank: XM_005655350.3). Subsequently, *EcoR I* and *Xho I* restriction enzymes were employed to perform double digestion on the amplified products and overexpression vectors. The recombinant overexpression plasmid pcDNA3.1-3Flag-C-MST2 was generated by T4 DNA ligase (Takara, Tokyo, Japan) and transfected into PK-15 cells with Lipofectime3000 (Invitrogen, Waltham, MA, USA). Expression of MST2 was assessed using WB and IFA.

#### Construction of *MST2* gene knockout PK-15 cell line

Three sets of single guide RNA (sgRNA) sequences, incorporated *Bsmb I* cleavage site, targeting MST2 gene, namely, sgRNA79, sgRNA15, and sgRNA8 (Table S1), were designed by the CHOPCHOP (http://chopchop.cbu.uib.no/). Restriction enzyme *Bsmb I* was utilized to linearize lenti-CRISPRV2 vector, which were then ligated to sgRNAs through gradient annealing PCR. Following transfection into PK-15 cells with the recombinant knockout plasmids, positive knockout cells were selected by puromycin of 2.5 μg/mL. Afterward, knockout cells were subcloned utilizing a limited dilution. Once the monoclonal cells had been cultured, genomic DNA was extracted, and the mutant site regions were identified by PCR amplification and sequencing. The knockout efficiencies of three groups of sgRNAs were evaluated through real-time qPCR and WB, respectively. Ultimately, the cells exhibiting the highest knockout efficiency were designated as MST2-KO.

#### Intracellular proliferation of *T. gondii*

PK-15 wild-type and MST2-KO cells were incubated with 1000 tachyzoites for 3 h under normal growth conditions (37°C, 5% CO2). Free parasites were washed away by PBS and the cells were further incubated 21 h. The cells were then fixed with 4% paraformaldehyde for 30 min and permeabilized with 0.3% Triton X-100 in PBS for 30 min at room temperature. After washing by PBS three times, the cells were blocked with 3% BSA for 30 min at room temperature. Then, the cells were incubated with mouse polyclonal anti-GRA7 for 1 h at room temperature. After washing by TBST five times, the cells were incubated with FITC-conjugated goat anti-mouse IgG for 1 h at room temperature and washed by TBST five times. The tachyzoites were counted in at least 100 PVs, and the results are representative of three independent experiments.

#### Regulatory roles of MST2 on apoptosis and hippo signaling pathway in PK-15 cells during *T. gondii* infection

FCM and WB were used to investigate the effects on apoptosis of PK-15 cells with MST2 overexpression or knockout, respectively. While real-time qPCR and WB were utilized to evaluate the influences on expressions of LATS1 and YAP of Hippo signaling pathway in PK-15 cells with MST2 overexpression or knockout, respectively. To assess the effects of MST2 knockout on apoptosis and Hippo signaling pathway activation in PK-15 cells during *T. gondii* infection, the infected normal and MST2-KO cells were collected, respectively. Regulatory roles of MST2 knockout on apoptosis and expressions of Hippo signaling pathway in *T. gondii*-infected PK-15 cells were evaluated by FCM, real-time qPCR and WB.

### Quantification and statistical analysis

#### Statistical analysis

In this study, statistical data were representative of ≥3 independent experiments and presented as the mean ± standard deviation (SD). Comparisons between two groups were analyzed using the t-test, while comparisons among multiple groups were analyzed using one-way analysis of variance (ANOVA). Differences were considered statistically significant at a *p* value of <0.05. All statistical analyses were carried out using GraphPad Prism 8.0 software (GraphPad Software, San Diego, CA, USA).
