# Supplementary material for: Toxoplasma gondii induces MST2 phosphorylation mediating the activation of hippo signaling pathway to promote apoptosis and lung tissue damage
Source: iScience. 2024 Nov 4;27(12):111312. doi: 10.1016/j.isci.2024.111312 (PMC11618000; doi:10.1016/j.isci.2024.111312)
Supplement: Document S1. Figures S1 and Table S1 [file mmc1.pdf]

iScience, Volume *27*

Supplemental information

Toxoplasma gondii induces MST2 phosphorylation mediating the activation of hippo signaling pathway to promote apoptosis and lung tissue damage

Kangzhi Xu, Shifan Zhu, Fan Xu, Jin Yang, Bin Deng, Dingzeyang Su, Jing Ma, Mingyue Zu, Yifan Lin, Tianxu Pei, Yuyang Zhu, Lele Wang, Dandan Liu, Qiangde Duan, Jinjun Xu, Zhiming Pan, Jianping Tao, and Zhaofeng Hou

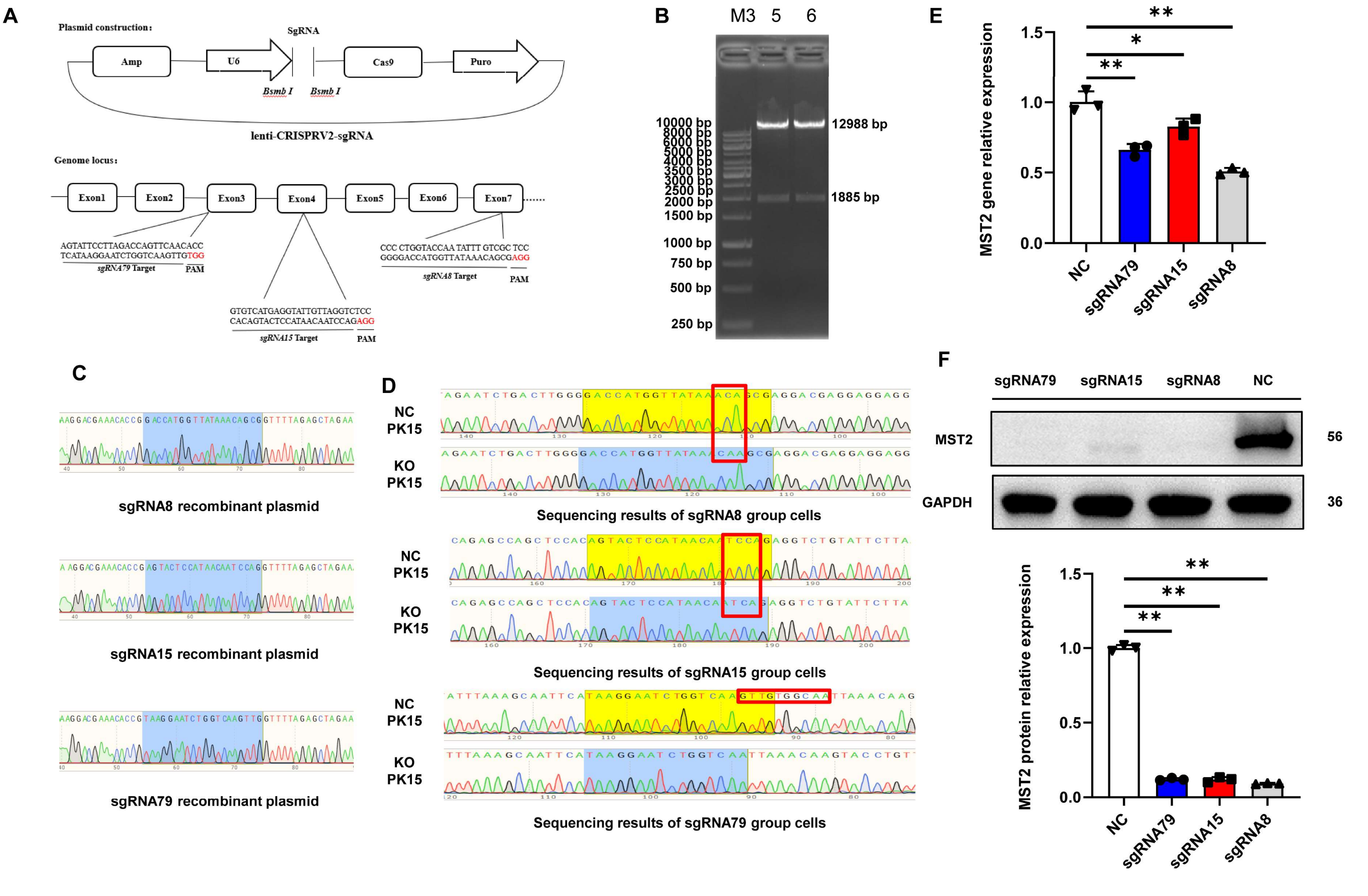

Figure S1. Construction of MST2 knockout cell lines and evaluation of knockout efficiency, related to Figure 6, 7. (A) Pattern diagram of MST2 knockout plasmid construction. (B) Knockout plasmid lenti-CRISPRV2 was linearized by *Bsmb I* enzyme. M3: 1kb DNA ladder; 5,6: lenti-CRISPRV2 was linearized. (C) The peak map of the sequencing of the recombinant knockout plasmid lenti-CRISPRV2-sgRNA8, lenti-CRISPRV2-sgRNA15, lenti-CRISPRV2-sgRNA79. (D) The sequencing map of the mutated region in MST2 knockout cells is compared to the unmutated region in normal cells. (E) qPCR assessment of MST2 knockout efficiency (n=3). (F) WB assessment of MST2 knockout efficiency (n=3). All graph data are expressed as the mean ± SD of at least three biological replicates per group. **P*< 0.05, ***P*< 0.01, ns, not significant.

Table S1 Sequences used in the experiments, related to Figure 4, 5, 6, S1.

Name	Sequence (5' to 3')
MST2-F(*EcoRI*)	CGGAATTCATGGAGCAGCCGCCGGC
MST2-R(*XhoI*)	CCCTCGAGTCAAAAGTTCTGCTGCCTTC
sgRNA79-F	CACCGTAAGGAATCTGGTCAAGTTG
sgRNA79-R	AAACCAACTTGACCAGATTCCTTAC
sgRNA15-F	CACCGAGTACTCCATAACAATCCAG
sgRNA15-R	AAACCTGGATTGTTATGGAGTACTC
sgRNA8-F	CACCGGACCATGGTTATAAACAGCG
sgRNA8-R	AAACCGCTGTTTATAACCATGGTCC
qPCR-MST2-F-pig, mice	TGGCTGACATCTCGTCCCTTGG
qPCR-MST2-R-pig, mice	TCCTGAATGTTGGTGGTGGGTTTG
qPCR-LATS1-F-pig	GCCAGCACCAACGATGTCTAGC
qPCR-LATS1-R-pig	ATGACACCAACACTCCACCAATCAC
qPCR-LATS2-F-pig	CTCCTTCAACAGCCACCAGCAG
qPCR-LATS2-R-pig	CTCAGCACCCGCACACTCTTG
qPCR-YAP-F-pig	TTCACAGCAGAACCGTTTCC
qPCR-YAP-R-pig	AAGCTTCTTGCAGACTTGGC
qPCR-TAZ-F-pig	CATCACGGTCTCCAATCACCAGTC
qPCR-TAZ-R-pig	TCCAACGCATCAACTTCAGGTTCC
qPCR-TEAD1-F-pig	TCAGGACAGGGAAGACGAGGAC
qPCR-TEAD1-R-pig	ACTAGACACCTTAATGGCGGCTTG
qPCR-GAPDH-F-pig	GTCGGTTGTGGATCTGACCT
qPCR-GAPDH-R-pig	CTTGACGAAGTGGTCGTTGA
qPCR-LATS1-F-mice	CTCACAGGCGGATGTAGGAAGAC
qPCR-LATS1-R-mice	TCGGAGGTGGTGGAGGAGTAAC
qPCR-LATS2-F-mice	CACAGCACCTGCCACTGAGAG
qPCR-LATS2-R-mice	GAGCCGCCATAGTCCACATCC
qPCR-YAP-F-mice	ACTCCGAATGCAGTGTCTTCTCC
qPCR-YAP-R-mice	CCGCTGTCTGTGCTCTCATCTC
qPCR-TAZ-F-mice	CACCGTCTCCAACCACCAGTC
qPCR-TAZ-R-mice	CAACGCATCAACTTCAGGTTCCAG
qPCR-TEAD1-F-mice	ATGAGCGACTCGGCAGATAAGC
qPCR-TEAD1-R-mice	CCCACACGGCGGATAGATAGC
qPCR-GAPDH-F-mice	TCTCCTGCGACTTCAACA
qPCR-GAPDH-R-mice	TGTAGCCGTATTCATTGTCA

Note: The MST2 sequence is marked with underlined *EcoR I* and *Xho I* cleavage sites, while the sgRNA sequences are also marked with underlined *Bsmb I* cleavage sites.
